# Atlantic salmon (*Salmo salar*) aquaculture in a changing climate: Quantitative insights from industry records and climate projections

**DOI:** 10.1111/jfb.70655

**Published:** 2026-09-20

**Authors:** Travis E. Van Leeuwen, David Coté, Cassandra Konecny, Andrew Swanson, Julia Bungay, Christina Pretty, Mark D. Fast, Jordan Miller, Trond Kristiansen

**Affiliations:** ^1^ Fisheries and Oceans Canada St. John's Newfoundland Canada; ^2^ Cooke Aquaculture Inc. Saint John New Brunswick Canada; ^3^ Cooke Aquaculture Inc. St. Alban's Newfoundland Canada; ^4^ Atlantic Veterinary College, University of Prince Edward Island Charlottetown Prince Edward Island Canada; ^5^ Actea Inc. San Francisco California USA; ^6^ Farallon Institute Petaluma California USA

**Keywords:** aquaculture, climate change, modelling, Newfoundland and Labrador, *Salmo salar*, salmonids

## Abstract

Predicting and understanding the implications of climate change on Atlantic salmon (*Salmo salar*) aquaculture is a fundamental step to understanding risk and mitigating potential impacts. Here, we statistically downscale established climate models using satellite‐based sea surface temperature data and in situ industry measurements to inform climate resiliency predictions for Atlantic salmon aquaculture on the south coast of Newfoundland, Canada. Our objectives were to (1) understand how local climate conditions and depth effects vary in the coastal marine environments that support farmed Atlantic salmon production; (2) apply these relationships to understand how seasonal chronology and forecasted interannual changes to water temperature will affect future farmed Atlantic salmon growth and sea lice (*Lepeophtheirus salmonis*) exposure; and (3) identify the seasonal mortality risk to farmed Atlantic salmon smolt under multiple climate change and sea lice scenarios. While the absolute growth of farmed Atlantic salmon was determined to be minimally affected, the chronology of growth changed with optimal growth periods predicted at deeper sea cage depths (10 and 15 m), and shifted from unimodal growth distributions throughout the summer to scenarios with two distinct growth peaks bookending a low summer growth period for 1 and 5 m depths as the time series progressed. Warm summer temperatures also increased the projected abundance of sea lice and decreased farmed Atlantic salmon smolt survival. Our results identify both resilience and vulnerabilities in Newfoundland Atlantic salmon aquaculture sites and highlight the risk to industry and an opportunity to mitigate vulnerabilities prior to their predicted onset.

## 1 INTRODUCTION

For the first time in 2022, aquaculture‐produced protein surpassed that acquired from wild capture fisheries, whose production has plateaued in recent decades (FAO, 2024). The expanding human population will create further demand that will need to be supplied by the aquaculture industry, particularly if wild fisheries are further compromised by sustainability issues and climate disruptions. What is less certain is how the existing global aquaculture industry is impacted by climate change (Froehlich et al., 2018).

Anthropogenic greenhouse gas emissions continue to increase and are predicted to raise global air temperature by 1.8–4.0°C by the year 2100 (Eyring et al., 2016; Hein et al., 2012; O'Neill et al., 2016; Tebaldi et al., 2021). This has already resulted in profound changes and disruptions to the physical environment and ecosystem that are expected to increase (Kwiatkowski et al., 2020; Scheffers et al., 2016). Some examples of changes to the physical environment are increased ocean temperature, rising sea level, more frequent extreme weather events, acidification and changes in rainfall (Geissinger et al., 2024; von Biela et al., 2022). Industries such as Atlantic salmon, *Salmo salar*, Linnaeus, 1758 aquaculture, that have production phases exposed to ambient weather and environmental conditions, can suffer losses caused by mortalities, escapes, disease, delayed production cycles and quality of fish at harvest (Calado et al., 2021; De Silva & Soto, 2009; Wade et al., 2019). For example, farmed Atlantic salmon are often raised in temperature‐controlled freshwater hatchery facilities from egg to smolt and transferred to open‐net pens in coastal marine environments, where, in Newfoundland, Canada, they are grown for 18 months until harvest. During this time, the farmed salmon are dependent on commercially produced pelleted feed, veterinary care and ambient environmental conditions to provide sufficient growth and survival to make production operations viable. Most fishes, including Atlantic salmon, are poikilotherms and hence cannot regulate their body temperature. Therefore, physiological function and metabolic rate are directly influenced by environmental temperature fluctuations (Brett, 1971). The relationship between water temperature and Atlantic salmon growth is parabolic, but optimal ranges are generally between 11 and 14°C (Falconer et al., 2020; Handeland et al., 2008). Water temperatures that are too warm (>20°C; Falconer et al., 2020) or cold (<7°C; Falconer et al., 2020; Sandrelli et al., 2025) can result in cessation of growth, reduced welfare, accelerated or delayed production cycles and mortality (Sandrelli et al., 2025; Wade et al., 2019). Additionally, while optimal water temperature conditions are favourable for salmon growth and production, they can also accelerate the development of the salmon louse (*Lepeophtheirus salmonis*, Krøyer, 1837; Godwin, Fast, et al., 2020; Rittenhouse et al., 2016; Sandvik et al., 2021), a naturally occurring and widespread parasite of Atlantic salmon (Young et al., 2026). This can result in an increase in the rate of salmon infestation and synergistically contribute to economic losses through higher mortality of cultured salmon and by increased use of expensive sea lice treatments (Hamoutene et al., 2022). Sandvik et al. (2021) found that an increase in water temperature from 9 to 11°C resulted in a two‐fold increase in total sea lice infestation pressure. Similarly, Godwin, Fast, et al. (2020) found that the effects of sea lice on the growth rate, condition and survival of Atlantic salmon smolt all synergistically worsened with increasing temperatures from 10 to 22°C.

Water temperature extremes, both high (Burke et al., 2020; Gamperl et al., 2021) and low (Sandrelli et al., 2025), in addition to sea lice infestation, have resulted in several mortality events (>10% mortality at a cage site) on farms along the south coast of Newfoundland in recent years. Prolonged periods of elevated sea surface temperature (SST) (e.g., 18–21°C) in combination with reduced oxygen saturation (approximately 70%) were suggested as the major drivers of mortalities. Notably, in early September 2019, such events resulted in total mortality in some sea cages, although there are conflicting views as to whether natural variation in environmental factors (e.g., water temperature and oxygen) was solely responsible, as Atlantic salmon were concurrently being stressed by sea lice treatments (Burke et al., 2020; Gamperl et al., 2021). Similarly, in 2025, more than 900,000 Atlantic salmon across five Newfoundland sites perished due to combined environmental, sea lice and treatment‐related stress factors.

Predicting and understanding the implications of climate change on Atlantic salmon aquaculture is a fundamental step to understanding risk (e.g., environmental, financial, etc.) and mitigating impacts (Froehlich et al., 2018). Climate models are a well‐used tool to define the expected change associated with various carbon emission scenarios. Global models can be further calibrated to local environments using downscaling techniques that correct predictions to local conditions using empirical data and an underlying understanding of physical processes. In marine environments, the poor resolution (approximately 100 × 100 km) of climate models and downscaling techniques have prevented their application in constrained areas with high variability in habitat characteristics that occur across small scales (e.g., coastal bays and inlets). Accounting for depth effects on water temperature further complicates projections and has typically been overlooked in the literature. As such, it has been difficult to accurately apply global climate projections to the environments where industries such as Atlantic salmon aquaculture typically operate (Falconer et al., 2020).

Here, we statistically downscale established coupled model intercomparison project 6 (CMIP6) climate models using high‐resolution satellite‐based SST data and in‐situ industry measurements that vary temporally and vertically across operational cage depths to inform climate resiliency predictions for Atlantic salmon aquaculture on the south coast of Newfoundland. Our objectives were to (1) understand how local climate conditions and depth effects vary in the coastal marine environments that support farmed Atlantic salmon production; (2) apply these relationships to understand how seasonal chronology and forecasted interannual changes to water temperature will affect future farmed Atlantic salmon growth and sea lice (*L. salmonis*) exposure; and (3) identify the seasonal mortality risk to farmed Atlantic salmon smolt under multiple climate change and sea lice scenarios.

## 2 METHODS

### 2.1 Study region and aquaculture production

The south coast region of insular Newfoundland (Figure 1a,b) is characterized by deep fjords and moderate tidal currents that vary considerably throughout the season (Donnet et al., 2022; Ratsimandresy et al., 2019). Oceanographic conditions are driven primarily by large‐scale oceanic circulation via the inshore branch of the Labrador Current, but wind forcing, bathymetry and advective transport are also important (Ratsimandresy et al., 2019). SSTs in recent years have generally been above zero in winter months, with rapid warming occurring during May to September when SSTs can exceed 20°C. Thereafter, a gradual decline in temperature occurs throughout the fall. An SST composite index from northern Labrador to the Scotian Shelf indicates a general warming trend throughout the northwest Atlantic since the late 1990s (Cyr et al., 2024). Salinity within the upper water column is highly variable and ranges from <25 psu in the inner Bay d'Espoir fjord, 25–31 psu in the middle and outer Bay d'Espoir fjord, where steelhead trout (*Oncorhynchus mykiss*) aquaculture is primarily conducted, and 30–33 psu in Little Passage, Hermitage Bay, Fortune Bay and Placentia Bay where Atlantic salmon aquaculture primarily occurs (Anderson et al., 2005; Dempson et al., 2024; Murray & Ang, 2018; Pepper et al., 2003).

**FIGURE 1:**
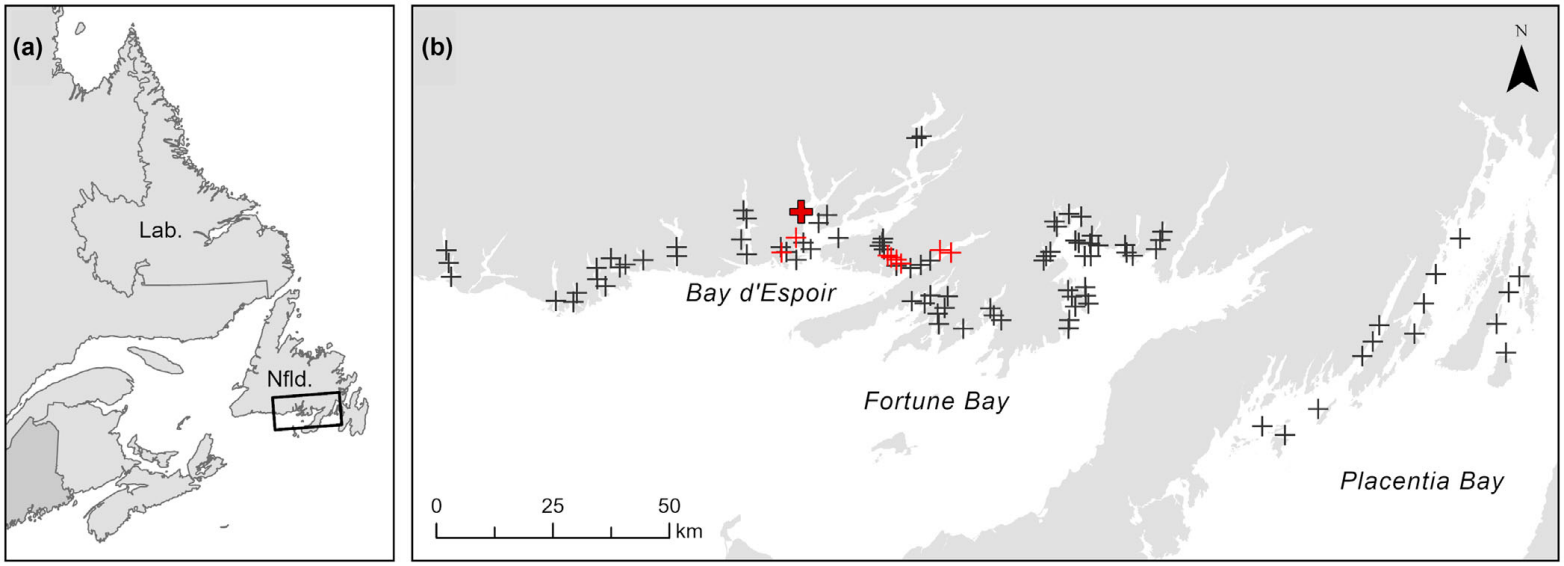
The region of Atlantic salmon aquaculture in relation to insular Newfoundland, Canada (a) and lease sites (crosses) designated for Atlantic salmon aquaculture (b). Lease sites in red are those in which industry‐provided water temperature data, with the bold red lease site (Sugarloaf), being the one in which analyses were focused. Not all lease sites are used within a given year.

On the south coast of Newfoundland, salmonid aquaculture (Atlantic salmon and steelhead trout) production has been modest (~8000–19,000 t annually; Dempson et al., 2024), but has increased to approximately 23,000 t in recent years. Nevertheless, the region has experienced considerable expansion from its start in Bay d'Espoir in the mid‐1980s, including industry growth east into Fortune Bay (Anderson et al., 2005; Pepper et al., 2003) and Placentia Bay (DFO, 2022) and more recently westward to Bay de Vieux (DFO, 2024), with further interest expressed from industry to expand westward.

### 2.2 Site‐specific industry water temperature data

Average daily water temperature data at 1, 5, 10 and 15 m depths were supplied by Cooke Aquaculture Inc. for nine Atlantic salmon aquaculture sites in our study region (Figures 1b and 2). Time series of data ranged from 2013 to 2023 with daily averages that typically spanned the entire production cycle (~18 months). However, data were only available for periods in which the sites were stocked with fish (Figure 2).

**FIGURE 2:**
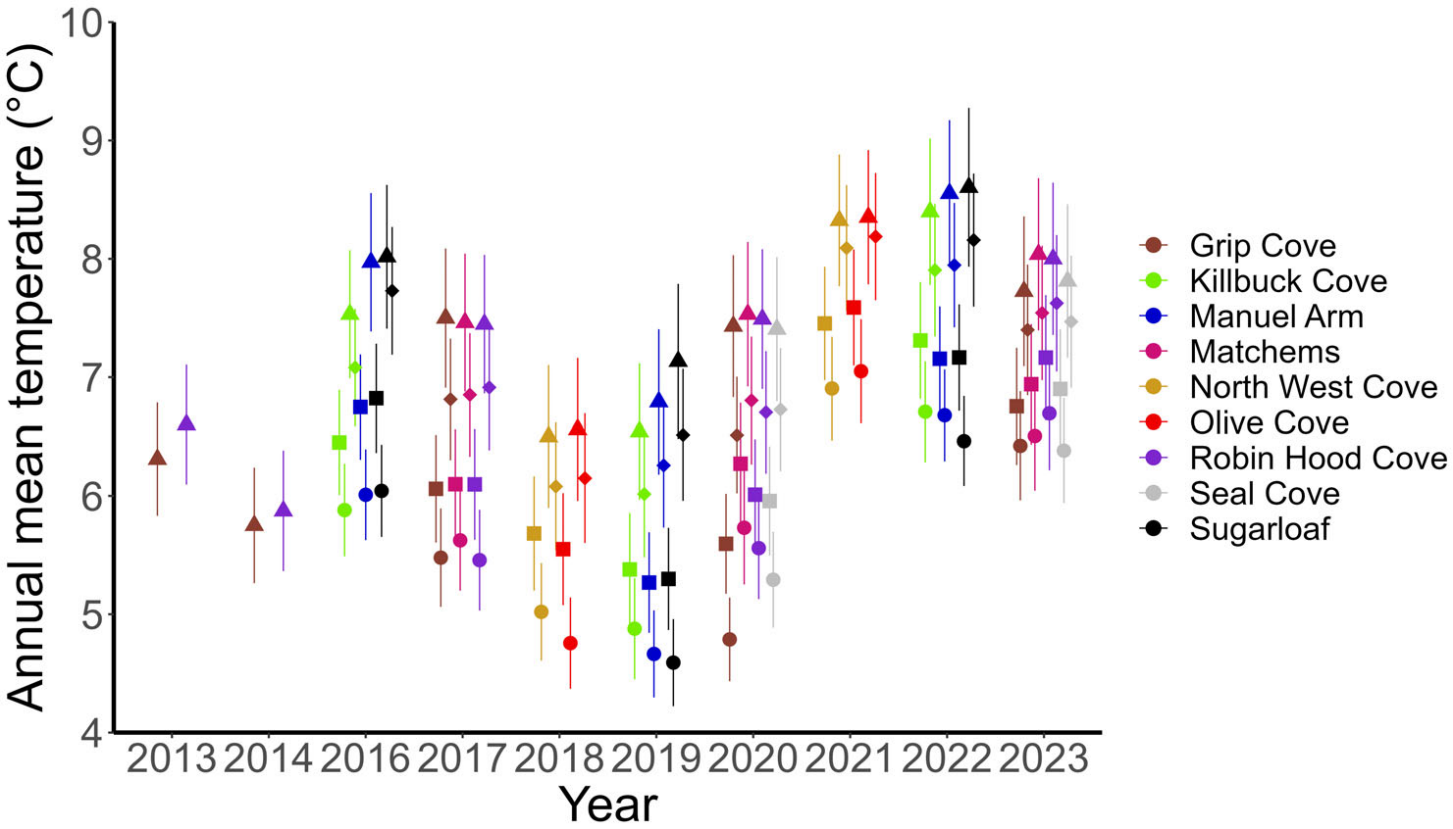
Annual mean water temperature at 1 m (triangle), 5 m (diamond), 10 m (square) and 15 m (circle) depths and year for various Atlantic salmon aquaculture sites located on the south coast of Newfoundland, Canada. Error bars represent 95% confidence intervals.

### 2.3 Climate models and climate projections using satellite‐based statistical downscaling

Climate models are used as a primary means to quantify past climate changes and extend that understanding to project future trajectories. For the models used here, expected global greenhouse gas concentrations [emerging from the representative concentration pathways (RCPs)] under different shared socioeconomic pathways (SSPs) up to the year 2100 (O'Neill et al., 2016) were used as input. For the 6th Intergovernmental Panel on Climate Change (IPCC) report, five narratives provided alternative socio‐economic developments for the world, including sustainable development (SSP1), regional rivalry (SSP3), inequality (SSP3, SSP4), fossil‐fueled development (SSP5) and middle‐of‐the‐road development (SSP2). While in principle the two development streams of climate and socio‐economic scenarios are independent, not all combinations are feasible. For this study, the combinations SSP1‐RCP 2.6 and SSP2‐RCP 4.5 were selected.

An important part of the IPCC report is the output from the CMIP that simulates future climate using the described SSP narratives of future emission scenarios. The CMIP models are global in scope and cover the atmosphere, land and ocean, which provides information at a large scale. The relatively coarse‐scale resolution of these models (1 × 1‐degree longitude‐latitude in the ocean), however, does not adequately resolve details within the marine coastal domain. Therefore, to provide climate projections at a more regional level, we first applied a statistical bias‐correction (BC) followed by a statistical downscaling (SD) of four global climate model projections using detrended quantile mapping (DQM). The BC corrects systematic errors in the climate data to minimize the errors between observed and modelled values for a specific control period, while the SD allows for the establishment of a transfer function between the high‐resolution observation (MUR) and coarse resolution climate variables, which transform future large‐scale climate projections to local scales (Kristiansen et al., 2024). The BC and SD of CMIP6 climate projections to the regional level consisted of four steps: data preparation, BC based on observations, SD of the corrected data to higher resolution, and creation of ensemble averages from the downscaled models. Data preparation involved the raw data from general circulation models (GCMs) and earth system models (ESMs) represented on different global ocean grids, some with higher resolution in certain areas. To work consistently with these models, we interpolated the data to a uniform Cartesian grid of 0.5 × 0.5‐degree longitude/latitude using the Earth System Modelling Framework (ESMF) and the Python xesmf interface (Zhuang et al., 2023), allowing fast interpolation within a tested framework. The BC used DQM transformation to minimize errors between observed and modelled values for the years 2002–2023 (Kristiansen et al., 2024, 2026). The statistical bias (SB) was carried out at the resolution of the GCM or ESM, which is 0.5 × 0.5‐degree latitude/longitude. The SD step used SB‐corrected fields at 0.5 × 0.5 degrees latitude/longitude resolution in combination with multi‐scale ultra‐high resolution (MUR) SST satellite data from NASA (Chin et al., 2017) as input to the DQM transformation. The high‐resolution (~1 km × 1 km) MUR satellite observations cover the period 2002–2023 and represent state‐of‐the‐art thermal imaging. The resulting downscaled dataset is on a 1/100th degree latitude/longitude grid. The area‐integrated ensemble projections for the south coast of the Newfoundland region were defined as the domain 47.4° N to 47.8° N latitude and −56.2° E to −55.3° E longitude. Here, four different climate models for each scenario were statistically bias corrected and downscaled, including IPSL‐CM6A‐LR_r1i1p1f1, MPI‐ESM1‐2‐LR_r1i1p1f1, CMCCCM2‐SR5_r1i1p1f1 and MPI‐ESM1‐2‐LR_r2i1p1f1 for two scenarios, SSP1‐2.6 and SSP2‐4.5.

### 2.4 Local and depth corrections using industry data

We constructed a day of year‐based correction framework to adjust climate model SST projections to resolve temperatures at multiple depths (1, 5, 10, 15 m) for the study region. First, the daily industry‐supplied water temperature at 1 m depth was subtracted from the daily satellite‐based SST. Daily differences were then aggregated by day of the year to produce a day of the year climatology of mean SST correction. This represents the systematic bias between the industry and satellite SST data at 1 m depth. Similarly, to account for differences in temperature at other depths within the cage in which Atlantic salmon could occupy, we computed daily vertical temperature offsets relative to 1 m (5–1 m, 10–1 m, 15–1 m). These offsets were then converted to day of year and averaged across years to obtain a day of year‐based climatology of mean offsets for 5, 10 and 15 m depths. The day of year‐based SST and depth correction was then applied to the daily climate projections, yielding a ground‐truthed and depth‐resolved temperature value for each model, scenario and time step.

### 2.5 Calculation and statistical analyses

Annual mean, minimum and maximum water temperatures were calculated for each of the nine sites at 1, 5, 10 and 15 m depths for years in which mean daily water temperatures over the entire year were available. This was done to ensure that changes in water temperature were not simply due to more measurements occurring in cooler winter months or warmer summer months. Annual mean water temperature among sites, depths and years was then compared using a generalized linear model (GLM), with a Gaussian distribution, and annual mean water temperature included in the model as the response variable, and site, depth and year as independent variables. To compare how water temperatures across depths may change over the next century, the corrected day of the year climate projections at 1, 5, 10 and 15 m depths were averaged for three time periods based on the IPCC predictions. The ‘current’ or ‘now’ period was an average of industry‐supplied water temperatures from 2016, 2019 and 2022, whereas climate projection averages for the years 2041–2060 (IPCC mid‐term) and 2081–2099 (IPCC long‐term) were used as the mid and long‐term periods. All site‐level comparisons were conducted on the Sugarloaf aquaculture site in Bay d'Espoir, Newfoundland, which was chosen because of its central location to other Atlantic salmon aquaculture sites in Newfoundland and available industry data (Figure 1b). These data were then averaged across the various climate and socio‐economic scenarios for each model and used to calculate various growth and welfare‐related temperature thresholds, a temperature‐dependent Atlantic salmon relative growth index (RGI), sea lice indices and smolt survival probabilities under no lice, low lice (1.4 lice/fish) and high lice (6.8 lice/fish) scenarios.

Growth and welfare‐related temperature thresholds used were from Falconer et al. (2020), and included the number of days during which daily water temperatures were >20°C (warm‐stress), between 11 and 14°C (optimal) and <7°C (cold‐stress), in addition to the minimum and maximum water temperatures for each period. The number of days during which daily water temperatures were >20°C, between 11 and 14°C and <7°C were compared using a GLM, with a Poisson distribution and log‐link function, with each included in separate models as the response variable, and depth, period and their interaction as independent variables. Similarly, minimum and maximum water temperatures were also compared using a GLM with the same independent variables but with a Gaussian distribution.

The temperature‐dependent RGI for Atlantic salmon used a skewed normal distribution based on observations from fig. 1 in Collins et al. (2020). The RGI formula was as follows:

$$\mathrm{RGI}=\text{ifelse}\left(t<16,\exp\left(-1^{*}\;\left({\left(t-16\right)}^{2}\right)/25\right),\exp\left(-1^{*}\;\left({\left(t-16\right)}^{2}\right)/8\right)\right).$$

The relationship between the probability of observing sea lice on Atlantic salmon and water temperature was derived from Helland et al. (2015). In their multivariate model, temperature had a multiplicative effect on the contributions of other variables (see Figure 4 in their paper). Here, we fit a logistic regression to the temperature data in their Figure 3b to capture this temperature‐dependent risk‐multiplicative effect, which we call the sea lice indices. This function produced a similar curve to the temperature‐dependent sea lice infestation pressure model used by Sandvik et al. (2021), as seen in Figure 2 of their publication. Note that in Helland et al. (2015), the range of the curve as a function of temperature is from 4 to 18°C, and the fit of our logistic function extrapolates beyond this. The sea lice index formula used in our study was as follows:

$$\mathrm{Sea}\;\text{lice indices}=\left(1.018/\left(1+\exp\left(-0.217^{*}\;\left(t-10.655\right)\right)\right)+0.028\right).$$

**FIGURE 3:**
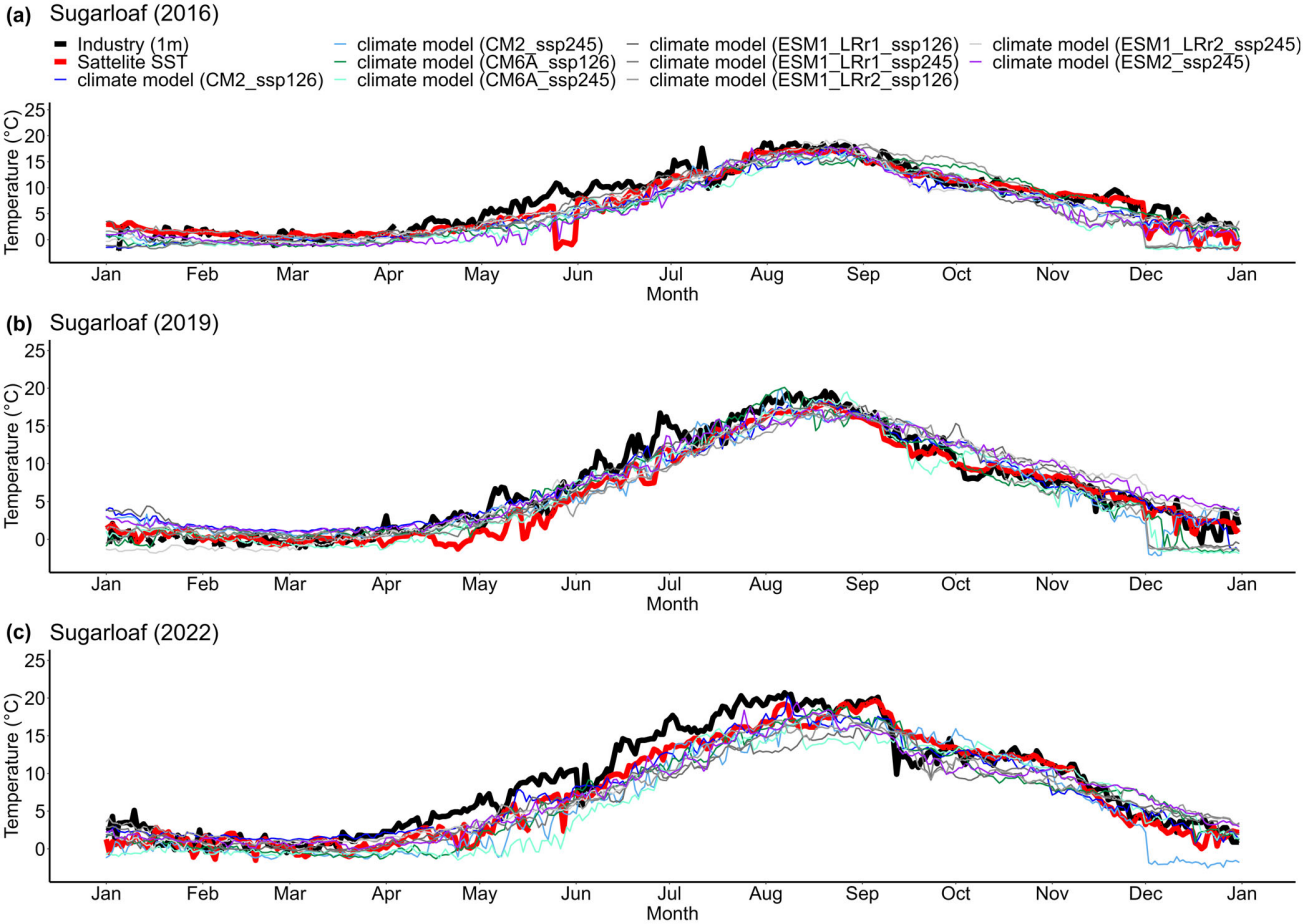
In situ industry water temperature measurements (black/bold) at 1 m depth in comparison to satellite‐based measurements of sea surface temperature (bold/red) and sea surface water temperature derived climate models for 2016 (a), 2019 (b) and 2022 (c), and used later for climate projections for the Sugarloaf production site in Bay d'Espoir, Newfoundland, Canada. Climate models were statistically bias corrected and downscaled, including IPSL‐CM6A‐LR_r1i1p1f1, MPI‐ESM1‐2‐LR_r1i1p1f1, CMCCCM2‐SR5_r1i1p1f1 and MPI‐ESM1‐2‐LR_r2i1p1f1 for two scenarios, SSP1‐2.6 and SSP2‐4.5.

Lastly, temperature‐dependent Atlantic salmon smolt survival models under no lice, low lice (1.4 lice/fish) and high lice (6.8 lice/fish) scenarios were derived from Godwin, Fast, et al. (2020) and are as follows:

$$\text{Survival}\left(\mathrm{no}\;\text{lice}\right)=\begin{array}{l} (\left(-0.000008^{*}\;t^{3}\right)+\left(0.0001^{*}\;t^{2}\right)+\left(-0.0016^{*}\;t\right)\\ +\;0.9966), \end{array}$$

$$\text{Survival}\left(\mathrm{low}\;\text{lice}\right)=\begin{array}{l} (\left(-0.00002^{*}\;t^{3}\right)+\left(-0.00002^{*}\;t^{2}\right)+\left(0.0006^{*}\;t\right)\\ +\;0.9909), \end{array}$$

$$\text{Survival(high lice)}=\begin{array}{l} (\left(-0.000005^{*}\;t^{3}\right)+\left(-0.0008^{*}\;t^{2}\right)+\left(0.0052^{*}\;t\right)\\ +\;0.9771). \end{array}$$

To examine relationships between Atlantic salmon RGI, sea lice indices and smolt survival probabilities under no lice, low lice and high lice scenarios, separate Beta regression models were used to evaluate significance at *p* < 0.05. Relationships between Atlantic salmon RGI and sea lice indices included period, depth and their interaction as factors, whereas the model for smolt survival probabilities under no lice, low lice and high lice scenarios included period, depth and sea lice scenario (i.e., no, low and high sea lice infestation) as factors.

Likelihood ratio tests, using the package lmtest (Zeileis & Hothorn, 2002), with and without covariates from the various models, were used to evaluate significance at *p* < 0.05 for the GLMs. All analyses, including summary statistics, Beta regressions and figures, were conducted using the statistical software R ver. 4.3.1 (R Core Team, 2023) and the packages dplyr (Wickham et al., 2023), betareg (Cribari‐Neto & Zeileis, 2010) and ggplot2 (Wickham, 2016), respectively. Maps were created using the software ArcGIS Pro ver. 3.3.2.

### 2.6 Ethics statement

This study did not involve the use of live animals. All analyses were based on previously published data and climate projections, and, therefore, no animal ethics approval was required.

## 3 RESULTS

### 3.1 Site‐level water temperature comparison

Daily maximum and minimum water temperatures across all nine sites were 22.0°C (Robin Hood Cove in 2023) and −2.1°C (Sugarloaf in 2016), respectively, with the warmest and coolest water temperatures occurring at 1 m depth (Table 1). There was no significant difference in average annual water temperatures among sites (GLM, *χ* ^2^ = −2.92, *p* = 0.47; Figure 2), suggesting that water temperatures experienced at a single site are representative of water temperatures across all nine sites (Figure 2). While there was no effect of site, there was a significant effect of depth and year on annual water temperatures, which increased across years (GLM, *χ* ^2^ = −18.43, *p* < 0.001; Figure 2) and decreased significantly with increasing depth (GLM, *χ* ^2^ = −45.43, *p* < 0.001; Figure 2).

**TABLE 1:** Water temperature extremes at 1, 5, 10 and 15 m depths and data range from the various Atlantic salmon aquaculture sites on the south coast of Newfoundland, Canada, with available data provided by industry.

Depth (m)	Metric	Grip Cove	Killbuck Cove	Manuel Arm	Matchems	North west Cove	Olive Cove	Robin Hood Cove	Seal Cove	Sugarloaf
	Data range (years)	2013–2017, 2019–2023	2015–2023	2015–2023	2016–2023	2015–2023	2015–2023	2013–2023	2015–2016, 2019–2020, 2022–2023	2015–2023
1	Temperature (min/°C)	−0.9	−0.6	−1.2	−1.0	−0.8	−1.3	−1.1	−1.2	−2.1
	Temperature (max/°C)	20.5	21.5	21.2	20.6	20.1	19.7	22.0	20.6	20.7
5	Temperature (min/°C)	−0.7	−0.5	−0.3	−0.7	−0.2	−0.3	−0.7	−0.7	−0.5
	Temperature (max/°C)	19.5	19.3	19.4	19.5	19.7	19.4	19.5	19.5	19.8
10	Temperature (min/°C)	0.1	−0.5	−0.1	0.1	0.6	0.1	0.1	0.1	0.0
	Temperature (max/°C)	17.3	19.1	17.4	18.6	19.0	19.4	19.0	17.9	18.9
15	Temperature (min/°C)	0.2	−0.4	0.2	0.1	0.9	0.2	0.9	0.3	0.4
	Temperature (max/°C)	17.1	17.7	16.1	17.8	17.2	17.4	18.0	16.8	17.6

### 3.2 Comparison of uncorrected modelled temperatures and industry measurements

Daily SSTs from the MUR satellite observations used in the climate projection models often underestimated the average daily water temperatures at 1 m depth supplied from industry for each of the 3 years (2016, 2019 and 2022) in which comparable data were available (Figure 3a–c). The daily offset ranged from −1.5°C (17 January) to 6.4°C (28 May), but 90% of the offsets were within −0.6 and 3.3°C. This underestimate was most pronounced during the spring warming period from April to July (Figure 3a–c). These systemic biases were accounted for in subsequent results (as outlined in the methods).

### 3.3 Potential effects of climate change on farmed salmon

While projected annual water temperatures significantly increased across all depths (GLM, *χ* ^2^ = −164.06, *p* < 0.001; Figure 4a–c) and periods (GLM, *χ* ^2^ = −69.14, *p* < 0.001; Figure 4a–c), the effect on farmed salmon differed depending on what depth water temperature measurements were used (Figure 4a–c). There was a significant interaction between period and depth on the number of days >20°C (GLM, *χ* ^2^ = −18.12, *p* < 0.001; Table 2). At 1 m depth the number of days >20°C increased from an average ± 95% CI of 3.0 ± 5.9 to 20.7 ± 2.6 and 33.6 ± 2.3 using the ssp126 and ssp245 models, respectively, for the period 2081–2099, whereas the number of days >20°C at 15 m depth did not change and was predicted to not increase above 20°C on any day for both the ssp126 and ssp245 models (Table 2; Figure 4a–c). Similarly, there was also a significant interaction between period and depth regarding the number of days between 11 and 14°C (GLM, *χ* ^2^ = −60.44, *p* < 0.001; Table 2). However, while the number of optimal temperature growth periods (Days 11–14°C) was not predicted to significantly change at 1 and 5 m depths, they more than doubled at 15 m depth, irrespective of the model used for the period 2081–2099 when compared to current values (GLM, *χ* ^2^ = −60.44, *p* < 0.001; Table 2). There was a significant effect of period and depth on the number of days <7°C. The number of days <7°C decreased irrespective of the model across time periods (GLM, *χ* ^2^ = −102.41, *p* < 0.001; Table 2) and for all depths (GLM, *χ* ^2^ = −163.75, *p* < 0.001; Table 2). Lastly, minimum and maximum water temperatures significantly increased irrespective of the model used across depths (Min. temperature GLM, *χ* ^2^ = −11.61, *p* < 0.001; Max. temperature GLM, *χ* ^2^ = −755.15, *p* < 0.001; Table 2) and periods (Min. temperature GLM, *χ* ^2^ = −32.72, *p* < 0.001; Max. temperature GLM, *χ* ^2^ = −120.65, *p* < 0.001; Table 2).

**FIGURE 4:**
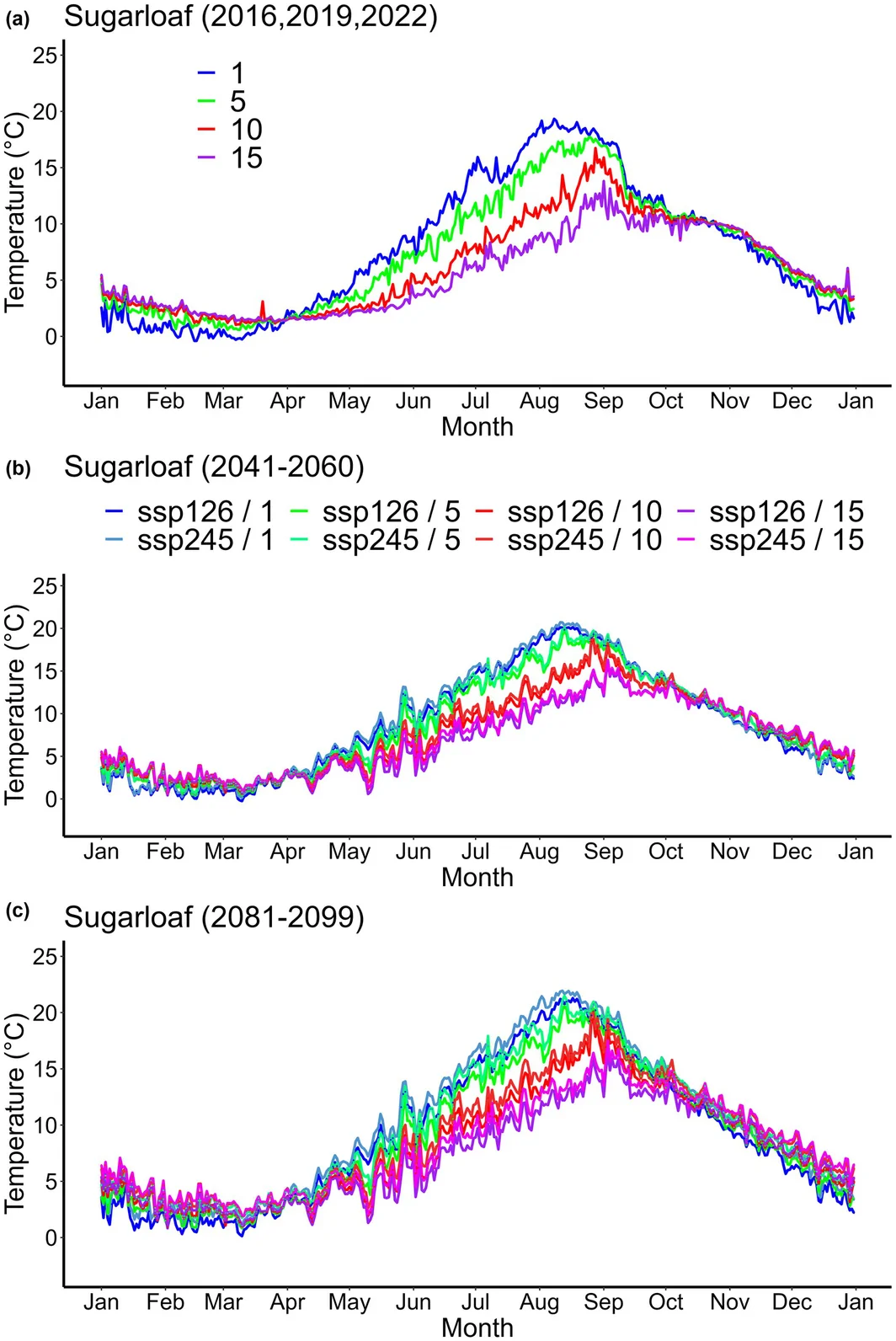
In situ (a; average of 2016, 2019 and 2022) and projected average measurements of water temperature at 1, 5, 10 and 15 m depths from the Sugarloaf aquaculture facility in Bay d'Espoir, Newfoundland, Canada, for the years 2041–2060 (b; mid‐term) and 2081–2099 (c; long‐term).

**TABLE 2:** Annual water temperature‐based thresholds ± 95% confidence intervals for Atlantic salmon growth and welfare, Atlantic salmon growth, sea lice burden and smolt survival under a no, low and high sea lice scenario at 1, 5, 10 and 15 m depths for now, 2041–2060 and 2081–2099 for the Sugarloaf Atlantic salmon aquaculture site on the south coast of Newfoundland, Canada.

Parameters			Growth and welfare‐related temperature thresholds					Atlantic salmon growth	Sea lice burden	Smolt survival		
Depth (m)	Period	Model	Days >20°C	Days 11–14°C	Days <7°C	Minimum temp (°C)	Maximum temp (°C)	Relative growth index	Sea lice index	No lice	Low lice	High lice
1	Now	Industry	3.0 ± 5.9	44.7 ± 10.1	168.0 ± 24.8	−2.1 ± 0.9	20.7 ± 1.2	0.24 ± 0.02	0.42 ± 0.03	0.94 ± 0.01	0.84 ± 0.03	0.73 ± 0.04
1	2041–2060	ssp126	9.4 ± 3.5	44.3 ± 3.1	169.0 ± 4.6	−1.6 ± 0.2	21.6 ± 0.3	0.26 ± 0.01	0.45 ± 0.01	0.94 ± 0.002	0.82 ± 0.01	0.70 ± 0.01
1	2041–2060	ssp245	15.2 ± 5.2	46.1 ± 2.7	160.3 ± 5.9	−1.3 ± 0.3	22.5 ± 0.4	0.27 ± 0.01	0.46 ± 0.01	0.93 ± 0.003	0.81 ± 0.01	0.69 ± 0.01
1	2081–2099	ssp126	20.7 ± 2.6	42.8 ± 3.5	160.7 ± 4.9	−1.3 ± 0.2	22.8 ± 0.3	0.25 ± 0.01	0.47 ± 0.01	0.93 ± 0.002	0.79 ± 0.01	0.66 ± 0.01
1	2081–2099	ssp245	33.6 ± 2.3	41.7 ± 3.3	146.1 ± 4.5	−0.3 ± 0.2	23.6 ± 0.3	0.26 ± 0.01	0.50 ± 0.01	0.92 ± 0.002	0.77 ± 0.01	0.64 ± 0.01
5	Now	Industry	0.0 ± 0.0	49.3 ± 19.5	177.0 ± 25.9	−0.5 ± 0.6	19.8 ± 1.1	0.24 ± 0.04	0.40 ± 0.04	0.95 ± 0.01	0.86 ± 0.02	0.76 ± 0.03
5	2041–2060	ssp126	1.8 ± 0.8	52.6 ± 4.0	175.2 ± 5.2	−0.7 ± 0.2	21.2 ± 0.4	0.28 ± 0.01	0.44 ± 0.01	0.94 ± 0.002	0.83 ± 0.01	0.72 ± 0.01
5	2041–2060	ssp245	4.0 ± 2.1	51.6 ± 3.6	164.8 ± 5.8	−0.5 ± 0.3	21.8 ± 0.4	0.28 ± 0.01	0.45 ± 0.01	0.94 ± 0.002	0.83 ± 0.01	0.71 ± 0.01
5	2081–2099	ssp126	6.4 ± 1.3	46.8 ± 3.1	165.7 ± 6.1	0.1 ± 0.1	22.3 ± 0.2	0.28 ± 0.01	0.46 ± 0.01	0.94 ± 0.002	0.81 ± 0.01	0.69 ± 0.01
5	2081–2099	ssp245	14.7 ± 3.0	43.8 ± 2.7	150.7 ± 4.2	0.5 ± 0.2	22.4 ± 0.2	0.29 ± 0.01	0.49 ± 0.01	0.93 ± 0.002	0.79 ± 0.01	0.66 ± 0.01
10	Now	Industry	0.0 ± 0.0	55.3 ± 33.5	202.7 ± 32.8	0.0 ± 0.6	18.9 ± 2.1	0.17 ± 0.06	0.35 ± 0.05	0.95 ± 0.01	0.88 ± 0.04	0.79 ± 0.06
10	2041–2060	ssp126	0.2 ± 0.2	60.8 ± 4.6	187.0 ± 5.5	−0.7 ± 0.2	20.7 ± 0.3	0.24 ± 0.01	0.40 ± 0.01	0.95 ± 0.002	0.85 ± 0.01	0.75 ± 0.01
10	2041–2060	ssp245	0.6 ± 0.5	64.6 ± 3.6	176.6 ± 5.7	−0.8 ± 0.3	20.9 ± 0.4	0.26 ± 0.01	0.41 ± 0.01	0.94 ± 0.002	0.85 ± 0.01	0.74 ± 0.01
10	2081–2099	ssp126	0.5 ± 0.2	59.7 ± 4.6	177.9 ± 5.8	0.0 ± 0.2	20.6 ± 0.2	0.26 ± 0.01	0.42 ± 0.01	0.94 ± 0.002	0.84 ± 0.01	0.72 ± 0.01
10	2081–2099	ssp245	1.8 ± 0.6	63.5 ± 3.9	161.2 ± 5.0	0.5 ± 0.2	22.0 ± 0.3	0.30 ± 0.01	0.46 ± 0.01	0.94 ± 0.003	0.82 ± 0.01	0.70 ± 0.01
15	Now	Industry	0.0 ± 0.0	35.7 ± 24.9	218.0 ± 39.6	0.4 ± 0.8	17.6 ± 2.3	0.10 ± 0.04	0.31 ± 0.04	0.97 ± 0.01	0.92 ± 0.04	0.85 ± 0.06
15	2041–2060	ssp126	0.0 ± 0.0	68.0 ± 4.5	196.0 ± 7.4	−0.9 ± 0.2	18.6 ± 0.4	0.18 ± 0.01	0.36 ± 0.01	0.96 ± 0.002	0.90 ± 0.01	0.82 ± 0.01
15	2041–2060	ssp245	0.0 ± 0.0	71.9 ± 4.8	182.5 ± 7.3	−0.5 ± 0.3	19.2 ± 0.4	0.20 ± 0.02	0.38 ± 0.01	0.96 ± 0.002	0.90 ± 0.01	0.81 ± 0.01
15	2081–2099	ssp126	0.0 ± 0.0	72.2 ± 5.5	181.7 ± 5.4	−0.2 ± 0.2	17.8 ± 0.2	0.21 ± 0.01	0.38 ± 0.01	0.96 ± 0.001	0.89 ± 0.004	0.81 ± 0.01
15	2081–2099	ssp245	0.0 ± 0.0	81.4 ± 4.6	166.2 ± 5.4	0.4 ± 0.3	19.4 ± 0.3	0.26 ± 0.01	0.42 ± 0.01	0.95 ± 0.002	0.88 ± 0.01	0.78 ± 0.01

There was a significant interaction between depth and period on absolute annual growth of farmed Atlantic salmon (betareg, *χ* ^2^ = 65.42, *p* < 0.001; Table 2; Figure 5a–c). While absolute annual growth was minimally affected at 1 and 5 m depths, it increased at 10 and 15 m depths (Table 2; Figure 5a–c). Interestingly, the chronology of growth changed with typical unimodal growth distributions throughout the summer continuing for deeper depths (10 and 15 m; Table 2; Figure 5a–c), and a shift at 1 and 5 m depths, from overlapping bimodal distributions in summer to distinct growth peaks bookending a low summer growth period, as the time series progressed through to the 2081–2099 period (Figure 5a–c).

**FIGURE 5:**
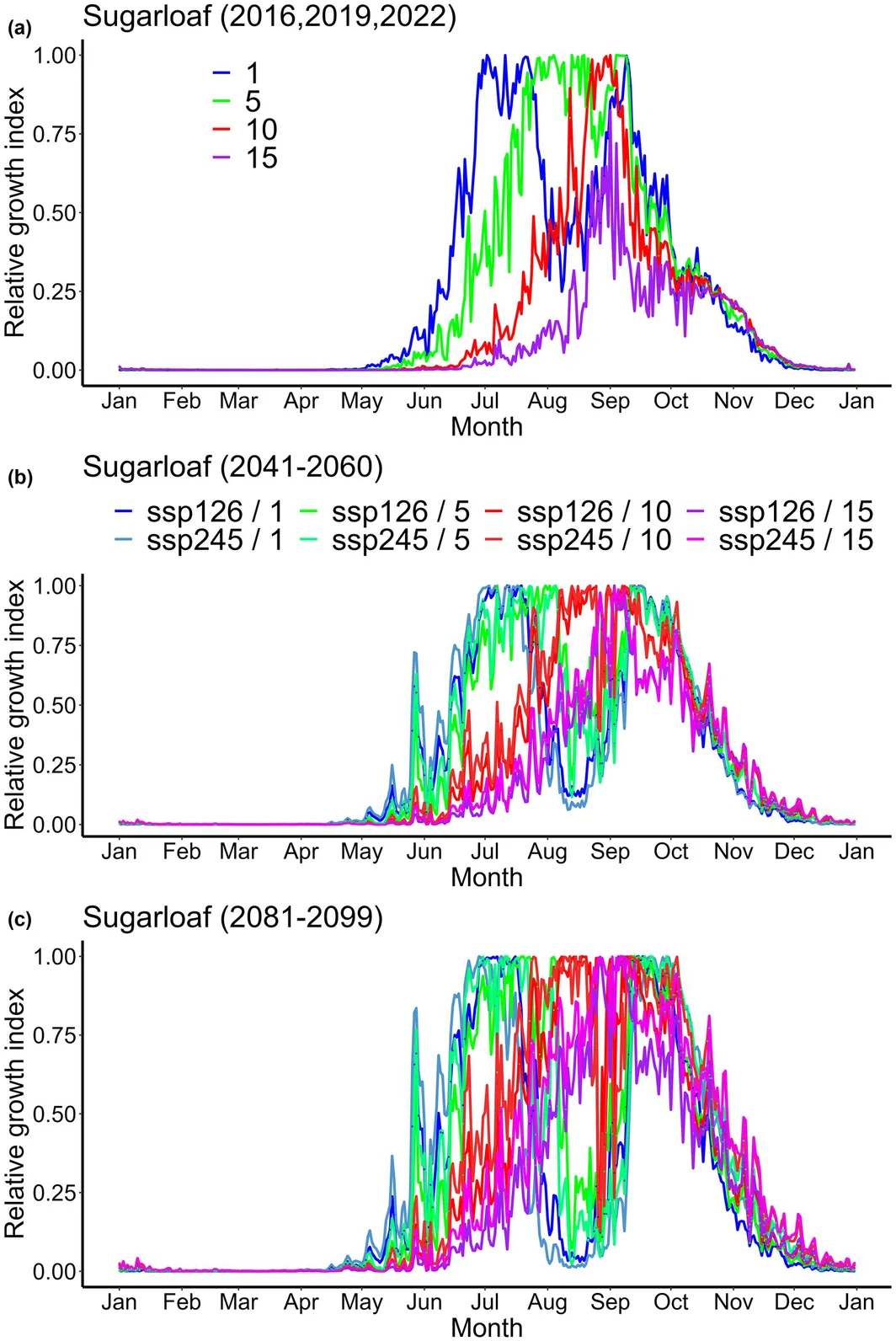
Current (a) and projected (b, c) average relative growth indices for Atlantic salmon derived from water temperature data at 1, 5, 10 and 15 m depths for the Sugarloaf aquaculture facility in Bay d'Espoir, Newfoundland, Canada, for the years 2041–2060 (b; mid‐term) and 2081–2099 (c; long‐term). The water temperature‐based equation for the Atlantic salmon growth indices was from Collins et al. (2020). Note the change from unimodal growth indices to bimodal growth indices using water temperatures at 1 and 5 m depths.

Warmer summer temperatures, however, significantly increased the projected threat of sea lice (betareg, *χ* ^2^ = 198.53, *p* < 0.001; Table 2; Figure 6a–c) at all depths (betareg, *χ* ^2^ = 604.45, *p* < 0.001; Table 2; Figure 6a–c) with the interaction between warmer summer temperatures and sea lice infestation level expected to significantly decrease Atlantic salmon smolt survival across time periods (betareg, *χ* ^2^ = 416.20, *p* < 0.001; Table 2; Figure 7a–i), especially at 1 and 5 m depths, depending on the lice infestation pressure (betareg, *χ* ^2^ = 2439.60, *p* < 0.001; Table 2; Figure 7a–i). Atlantic salmon smolt survival with no sea lice present remained similar across time periods and depths, ranging from 97% currently and water temperatures at a depth of 15 m to 93% and 92% using the ssp126 and ssp245 models, respectively, for the 2081–2099 period and water temperatures at a depth of 1 m (Table 2). However, under a high sea lice scenario, Atlantic salmon smolt survival decreased considerably, ranging from 85% currently when water temperatures at a depth of 15 m were used to 66% and 64% using the ssp126 and ssp245 models, respectively, for the 2081–2099 period and water temperatures at a depth of 1 m (Table 2).

**FIGURE 6:**
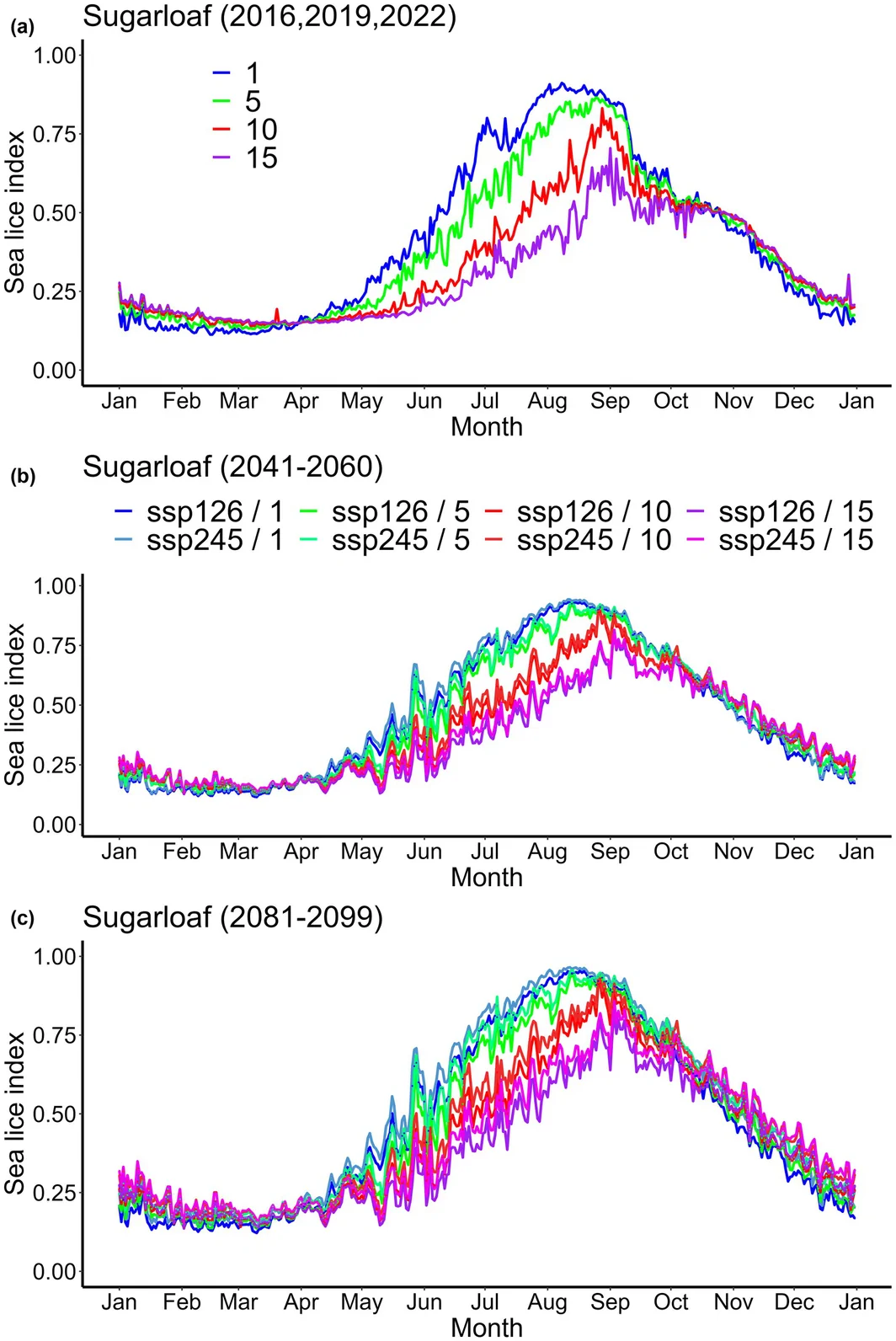
Current (a) and projected (b, c) sea lice indices derived from water temperature data at 1, 5, 10 and 15 m depths from the Sugarloaf aquaculture facility in Bay d'Espoir, Newfoundland, Canada, for the years 2041–2060 (b; Intergovernmental Panel on Climate Change [IPCC] mid‐term) and 2081–2099 (c; IPCC long‐term). The water temperature‐based equation for the sea lice indices was derived from (Helland et al., 2015).

**FIGURE 7:**
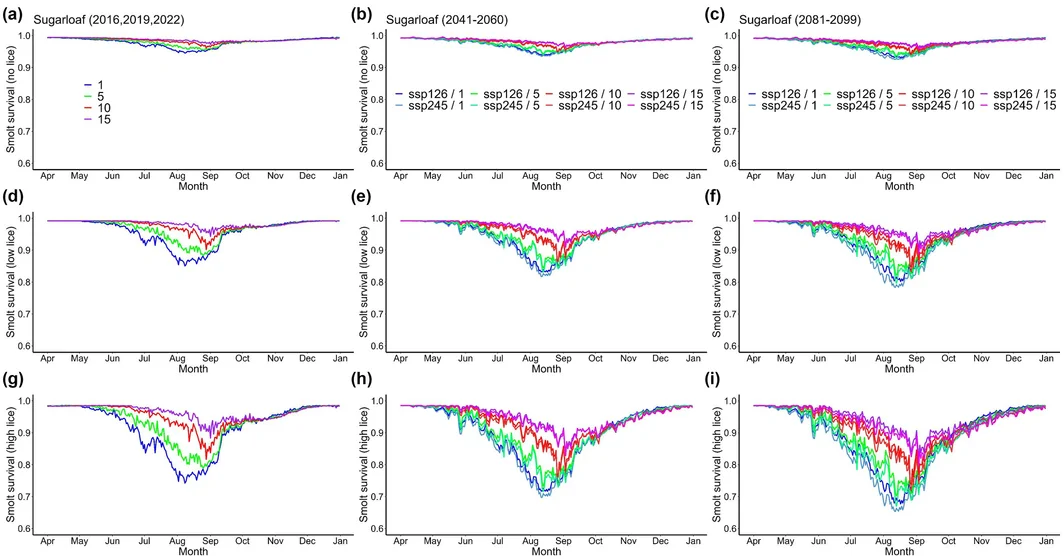
Survival probability of cultured Atlantic salmon smolt derived from water temperature data at 1, 5, 10 and 15 m depths under a no sea lice (a–c), low sea lice (average = 1.4 lice/fish; d–f) and high sea lice (average = 6.8 lice/fish; g–i) infestation scenarios for the Sugarloaf aquaculture facility in Bay d'Espoir, Newfoundland Canada, averaged for the years 2016, 2019, 2022 (a, d, g; current‐term), 2041–2060 (b, e, h; mid‐term) and 2081–2099 (c, f, i; long‐term). The water temperature‐based equations for the survival probability were derived from Godwin, Fast, et al. (2020).

## 4 DISCUSSION

Ecological and societal effects of climate change are complex and will vary geographically and by sector (Weatherdon et al., 2016). Compared to shellfish aquaculture, finfish aquaculture globally is expected to fare better in response to climate change (Froehlich et al., 2018), with suggestions that areas with existing farms should see increased growth rates as the climate warms under scenarios of sustainable or middle‐of‐the‐road development (Klinger et al., 2017). While a warming climate will create significant disruptions to marine ecosystems (Scheffers et al., 2016), our projections do not provide evidence that the annual growth of farmed Atlantic salmon in our study area on the south coast of Newfoundland will change considerably, as a result of warmer waters. While the absolute growth of cultured Atlantic salmon is predicted to be minimally affected, the chronology of production growth will likely change. Seasonality drives water temperatures and optimal growth periods shifted from somewhat unimodal growth distributions throughout the summer, to scenarios of distinct growth peaks bookending a low summer growth period as the time series progresses at the 1 and 5 m depths. Similar observations were made in wild gadids inhabiting nearshore marine areas (Laurel et al., 2017). Comparisons of populations inhabiting warm and cool nurseries showed bimodal and unimodal growth chronologies. However, an important distinction is that wild fish were able to relocate away from nurseries when water temperatures became too warm. Farmed fish will be restricted to the range of temperatures available to them within the depth ranges of their cage. Altered growth chronology could influence operations (e.g., time to market, etc.), but of greater concern are the stressors that farmed salmon could be exposed to during the warmer summer period.

Though absolute annual growth was not greatly affected by a warming climate at 1 and 5 m depths, it did increase considerably for the 10 and 15 m depths as a wider window of optimal growth days (i.e., 11–14°C) is predicted to occur at these depths. However, the number of temperature days >20°C, shown to be stressful and sometimes fatal to Atlantic salmon, is predicted to increase (6–10)‐fold over the next century and could result in increased frequency of mortality events, with maximum daily temperatures ±95% CI reaching 23.6 ± 0.3°C at 1 m depth. In early September 2019, such events resulted in total mortality in some sea cages (2.6 million Atlantic salmon across 10 sites; Gamperl et al., 2021). Similarly, in 2025, more than 900,000 Atlantic salmon across five sites perished due to environmental and sea lice‐related stress. Therefore, it is clear that a trade‐off will exist between sub‐optimal growth periods at 1 and 5 m depths versus an apparent improved growth potential at the 10 and 15 m depths. Gamperl et al. (2021) showed that farmed Atlantic salmon in Newfoundland predominantly used the upper few metres of the cage (<5 m) but did seek refuge in cooler, deeper water during a heat‐wave, although it is unclear whether stocking densities would allow all fish to occupy these more ambient areas of a sea cage.

Warmer summer temperatures, however, notably increased the projected threat of sea lice. Diseases and pathogens are expected to become more prevalent in aquaculture sites with climate change (De Silva & Soto, 2009; Godwin, Fast, et al., 2020; Godwin, Krkosek, et al., 2020; Mackintosh et al., 2025). Sea lice are already a burden on production sites in our study area (Dempson et al., 2024; Hamoutene et al., 2022; Young et al., 2026). Not only does warmer water in summer months favour the proliferation of sea lice by accelerating their life cycle (Godwin, Fast, et al., 2020), but warmer fall and winter temperatures may also result in prolonged windows of infestation risk and perhaps result in a greater capacity for gravid females to over‐winter and proliferate earlier into the spring (Beamish et al., 2007; Heuch et al., 2000). More frequent and longer duration windows for sea lice outbreaks may have consequences for both farmed salmon health, growth and survival, as well as for threatened wild Atlantic salmon populations from the south coast of Newfoundland that migrate through our study area (Dempson et al., 2024; Lilly et al., 2026). Therefore, sea lice management and mitigation measures will remain a priority for aquaculture and wild salmon conservation research.

The full extent of increased sea lice pressure is difficult to quantify. However, applying our climate projections to the smolt survival models of Godwin, Fast, et al., (2020), derived under controlled laboratory conditions, indicated important differences in Atlantic salmon smolt survival under various temperatures and elevated sea lice burden. For example, under high sea lice infestation, survival probabilities could drop below 70% during summer periods by the end of the century, whereas under no sea lice conditions, survival remains above 90%.

The adaptive nature of sea lice makes sea lice management and mitigation a continually evolving field. Current methods for controlling sea lice include the use of medicated feed (e.g., Slice®, IVOMEC®, Calicide®) individually, or in combination with bath treatments (Hamoutene et al., 2022) using azamethiphos (Salmonsan®), and hydrogen peroxide (Interox Paramove 50®), biological treatments using lumpfish or wrasse (Groner et al., 2013), and thermos‐mechanical methods using hydrolicers that pressurize water to dislodge sea lice from individual salmon, often in combination with warm water (Overton et al., 2018).

Besides selective breeding programs targeted at improving heat tolerance in Atlantic salmon (Gonen et al., 2024), a potential mitigation measure revealed by this study that could help mitigate against high temperatures and sea lice is to submerge cages to deeper depths (Dempster et al., 2008, 2009; Warren‐Myers et al., 2024). However, because Atlantic salmon are physostomous, providing adequate access to air to allow the salmon to fill their swim bladder will be necessary for long‐term holding and health. Several submerged cage technologies are being trialled for offshore locations that claim to address this issue (O'Neill et al., 2025). Nevertheless, based on our results, not only will the optimal growth window at deeper depths increase salmon growth and protect salmon from an increase in days >20°C, but it may also help alleviate sea lice burden by avoiding the depths (<5 m) in which the infective copepodid stage of *L. salmonis* is frequent (Bron et al., 1993). However, a recent study by O'Neill et al. (2025) suggests that sea lice from submerged cages produce larvae that swim deeper, suggesting that lice may become problematic at these deeper depths as well. The use of ‘skirted’ or ‘snorkel’ cages, which use an impermeable barrier around the cage to separate *L. salmonis* from the salmon in the surface waters inside the cage, where they are most abundant, may also help alleviate sea lice burden (Oppedal et al., 2019; Stien et al., 2018; Wright et al., 2017). An alternative method could be to limit or avoid the amount of time Atlantic salmon are held in cages where salinities are conducive for sea lice survival (i.e., >28 psu; Bricknell et al., 2006; although see Groner et al., 2016), although it is unclear how the lower salinity would affect salmon growth, flesh quality and taste, and whether this may drive sea lice towards increased tolerance (Ljungfeldt et al., 2017) or result in sea lice simply being replaced by other pests and pathogens such as *Ergasilus labracis*, Krøyer, 1863 which have been found in high densities in the upper part of Bay d'Espoir (Eaves et al., 2014). Salinity within the upper water column in Bay d'Espoir, Newfoundland is highly variable and ranges from <25 psu in the inner fjord, 25–31 psu in the middle and outer fjord, and 30–33 psu in Little Passage, Hermitage Bay, Fortune Bay and Placentia Bay, where most Atlantic salmon aquaculture is currently conducted (Anderson et al., 2005; Dempson et al., 2024; Murray & Ang, 2018; Pepper et al., 2003) and thus may be well suited for trialling such strategy.

Our method of combining high‐resolution SST data with industry in situ measurements provides a cost‐effective way to accurately downscale projections in the inshore habitats where aquaculture is prevalent, while still accounting for depth use by the salmon. While projections of absolute values should be used with caution, and can also differ considerably across climate models and scenarios (e.g., fossil‐fueled development; SSP5), relative differences (i.e., trends) across scenarios and the use of ensembles, as used in this study, are more robust. DQM preserves the CMIP6 projected trend in each quantile while correcting biases relative to a historical reference, but it assumes that the BC transfer function itself is stationary across the projection period. Consequently, structural changes in the relationship between modelled and true distributions under a warmer climate are not represented, which may attenuate the magnitude of projected extremes relative to a fully non‐stationary correction (Kristiansen et al., 2026).

Climate warming is also expected to come with increased risk of weather extremes. While our climate projections build in increased potential for extreme weather, the aggregate numbers reported here do not factor in the challenges of events like heatwaves, storms or extreme oceanographic events, including ‘super‐chill’ in the winter months (Sandrelli et al., 2025). Industry temperature monitoring has documented great and sudden changes in temperature (Sandrelli et al., 2025). For example, in July of 2025, a strong wind event created warm isothermal conditions in which cooler subsurface waters increased by over 12°C in a matter of hours.

Atlantic salmon aquaculture operations are a critical contributor to global protein production and an important driver of economic activity, particularly in the rural areas along the south coast of Newfoundland. At the same time, these operations are inherently vulnerable to ambient conditions that interact with farms in complex ways. In a changing climate, these complexities are compounded. Our results identify both climate change resilience and vulnerabilities in Atlantic salmon aquaculture operations along the south coast of Newfoundland. Our predictions provide industry and regulators a notice of risk, a window into how production efficiency is likely to shift in future years, and the opportunity to proactively develop appropriate mitigation solutions for future scenarios. Such mitigations will be important to safeguard wild fish, the aquaculture industry and the communities they support.

## AUTHOR CONTRIBUTIONS

**Travis E. Van Leeuwen:** Conceptualization; data collation; formal analysis; roles/writing—original draft; writing—review and editing. **David Coté:** Conceptualization; data collation; formal analysis; roles/writing—original draft; writing—review and editing. **Cassandra Konecny:** Data collation; writing—review and editing. **Andrew Swanson:** Conceptualization; data collation; writing—review and editing. **Julia Bungay:** Conceptualization; data collation; writing—review and editing. **Christina Pretty:** Data collation; writing—review and editing. **Mark D. Fast:** Conceptualization; data collation; writing—review and editing. **Jordan Miller:** Conceptualization; data collation; formal analysis; writing—review and editing. **Trond Kristiansen:** Conceptualization; data collation; formal analysis; writing—review and editing.

## FUNDING INFORMATION

No specific funding was provided for the study.

## CONFLICT OF INTEREST STATEMENT

Andrew Swanson and Julia Bungay are employees of Cooke Aquaculture Inc. Statistical downscaling was conducted by Actea Inc. (Jordan Miller and Trond Kristiansen) using patent‐pending methods. The remaining authors declare no conflicts of interest.

## Data Availability

All data generated or analysed during this study could be made available from the corresponding author upon request and permission of Cooke Aquaculture Inc.
